# Pharmacokinetics and pharmacodynamics of antibacterial peptide NZX in *Staphylococcus aureus* mastitis mouse model

**DOI:** 10.1007/s00253-024-13101-w

**Published:** 2024-03-13

**Authors:** Xueling Zheng, Na Yang, Ruoyu Mao, Ya Hao, Da Teng, Jianhua Wang

**Affiliations:** 1 Gene Engineering Laboratory, Feed Research Institute, Chinese Academy of Agricultural Sciences, 12 Zhongguancun Nandajie St., Haidian District, Beijing 100081 People’s Republic of China; 2 Innovative Team of Antimicrobial Peptides and Alternatives to Antibiotics, Feed Research Institute, Chinese Academy of Agricultural Sciences, Beijing, 100081 People’s Republic of China; 3 Key Laboratory of Feed Biotechnology, Ministry of Agriculture and Rural Affairs, Beijing, 100081 People’s Republic of China

**Keywords:** Antimicrobial peptide NZX, *Staphylococcus aureus*, Mechanism of action, Pharmacokinetics, Pharmacodynamics, Mouse mastitis model

## Abstract

**Abstract:**

*Staphylococcus aureus* is associated with dairy mastitis, which causes serious economic losses to dairy farming industry. Antibacterial peptide NZX showed good antibacterial activity against *S. aureus*. This study aimed to evaluate pharmacokinetics and pharmacodynamics of NZX against *S. aureus*-induced mouse mastitis. NZX exhibited potent in vitro antibacterial activity against the test *S. aureus* strains (minimal inhibitory concentration (MIC): 0.23–0.46 μM), low mutant prevention concentration (MPC: 1.18–3.68 μM), and a long post antibiotic effect (PAE: 2.20–8.84 h), which was superior to those of lincomycin and ceftiofur. Antibacterial mechanisms showed that NZX could penetrate the cell membrane, resulting in obvious cell membrane perforation and morphological changes, and bind to intracellular DNA. Furthermore, NZX had a good stability in milk environment (retention rate: 85.36%, 24 h) than that in mammary homogenate (47.90%, 24 h). In mouse mastitis model, NZX (25–400 μg/gland) could significantly reduce the bacterial load of mammary tissue in a dose-dependent manner. In addition, NZX (100 μg/gland) could relieve the inflammatory symptoms of mammary tissue, and significantly decreased its pathological scores. The concentration–time curve of NZX (100 μg/gland) in the mammary tissue was plotted and the corresponding pharmacokinetic parameters were obtained by non-compartment model calculation. Those parameters of T_max_, T_1/2_, C_max_ and AUC were 0.5 h, 35.11 h, 32.49 μg/g and 391 μg·h/g, respectively. Therefore, these results suggest that NZX could act as a promising candidate for treating dairy mastitis disease caused by *S. aureus*.

**Key points:**

• *NZX could kill S. aureus by dual mechanism involved in membrane and DNA disruption*

• *NZX could relieve S. aureus-induced mouse mastitis*

• *Pharmacokinetic parameters of NZX in mouse mammary gland were obtained*

## Introduction

In livestock production, dairy mastitis caused by *Staphylococcus aureus* (*S. aureus*) is the most frequent disease of dairy cattle (Ruegg 2017). According to the clinical characteristics of diseases symptoms, dairy mastitis can be divided into two major subtypes: clinical mastitis and subclinical mastitis (Krishnamoorthy et al. 2021). Clinical mastitis was defined by abnormal changes in consistency of milk, or the presence of skin signs of inflammation such as redness, swelling, heat or pain. Subclinical mastitis tends to persist because it usually remains undiagnosed (Krishnamoorthy et al. 2021). Dairy industry has confronted major economic losses due to discarded milk, medication use, labor costs, cow fatalities, as well as reproductive disorders (Nikkhah et al. 2021; Rainard et al. 2018; Sharma et al. 2017; Zouharova and Rysanek 2008). The current implementation into many farms of clinical practice guidelines on treatment of dairy mastitis is the intramammary antibiotics treatment. However, there are many risks in animals with antibiotics treatment, including potential drug residues in the human food supply, the emergence and dissemination of antimicrobial-resistance *S. aureus*, and economic losses associated with discarded milk (Vasquez et al. 2017; Xu et al. 2015). Therefore, there is an urgent need to find the ideal alternatives for antibiotic that could control *S. aureus* infections.

Antimicrobial peptide (AMPs) had those advantages of low resistance, rapid killing bacteria and no residues. In the past 60 years, the number of approved peptide drugs has grown steadily at an average growth rate of 7.7%, but it still faces many challenges in the development (Muttenthaler et al. 2021). The difficulties mainly included unstable clinical treatment effect, high cost, the difficulty of developing quantitative methods, stability issues and so on (Boix et al. 2017; Ewles and Goodwin 2011; Hoofnagle and Wener 2009; Jiang et al. 2021). NZX, a novel plectasin mutant, had been demonstrated to improved antibacterial activity against *Mycobacterium tuberculosis* and *S. aureus* due to its increase of net charge and hydrophobic moment (Tenland et al. 2018). In addition, NZX was expressed by *Pichia pastoris* X-33 conducted by Liu et al. (Liu et al. 2020), its total secreted protein levels were nearly 4 times higher than that of plectasin indicating its advantage in the preparation cost (Liu et al. 2020). Those merits along with low cytotoxicity, low resistance risk, high stability and good bactericidal activity against *S. aureus *in vitro and in vivo indicated that NZX had potential advantages of treating exudative epidermitis and mastitis caused by *S. aureus* (Liu et al. 2020; Zheng et al. 2022).

The studies of pharmacodynamics and pharmacokinetics of peptides will be the key links to further clinical application. In the exploration pharmacokinetics of plectasin, NZ2114 and NZX, a microbial assay, high performance liquid chromatography (HPLC) and high performance liquid chromatography-mass spectrometry (HPLC–MS) method for their quantitation in mouse serum, the drug serum concentrations-time curves and the responding parameters were established and obtained, which promoted the development of accurate quantitative detection technology of those peptides for moving closer to clinical application (Andes et al. 2009; Brinch et al. 2009; Zheng et al. 2022). However, pharmacodynamics and pharmacokinetics of NZX administered through the milk duct have not been studied so far. Based on structural similarity of mammary gland between mouse and bovine (Schmelcher et al. 2012; Wang et al. 2021; Yu et al. 2016), pharmacokinetics and pharmacodynamics of NZX administrated by milk duct against a mouse mastitis model infected with *S. aureus* were conducted in this study. In addition, the antibacterial characteristics, and antibacterial mechanisms of NZX against *S. aureus* strains were also studied in vitro. Through these studies, we hope to lay a theoretical and evaluation foundation for further clinical application of NZX of treating the dairy cow mastitis.

## Materials and methods

### Chemicals and strains

NZX (> 90% purity) was prepared as previous protocols (Liu et al. 2020). Lincomycin and ceftiofur were purchased from the Dalian Meilun Biotech (Dalian, China). *S. aureus* E48 (CGMCC 1.90006) isolated from bovine mastitis was provided by Professor Xin Zhao, Northwest A&F University (Yangling, China) and stored at the China General Microbiological Culture Collection Center (CGMCC). *S. aureus* ATCC 43300 and *S. aureus* ATCC 25923 were purchased from the American Type Culture Collection (ATCC).

### Model animal

The ICR female and male mice (7 to 8 weeks old) were purchased from Beijing Vital River Laboratories (Beijing, China).

### In vitro antibacterial characterization

Minimal Inhibitory Concentration (MIC), Minimal Bactericidal Concentration (MBC) and Mutant Prevention Concentration (MPC).

The MIC and MBC values of NZX against the test *S. aureus* strain were determined according to Clinical Laboratory Standards Institute (Watts et al. 2013). Briefly, a volume of 10 $\upmu$L diluted NZX with final concentration (1.25–1280 μg/mL) and 90 $\upmu$L of mid-log phase *S. aureus* cells (1 $\times$ 10^5^ CFU/mL) were added into a 96-well plate and co-incubated for 16–18 h at 37 °C. MIC value was defined as the lowest concentration of NZX at which no visible bacterial growth was observed. In addition, the bacteria suspensions were plated on Mueller–Hinton agar (MHA) medium to count, and the lowest concentration of NZX that results in 99.9% of died bacteria was the MBC value.

MPCs of NZX against the test *S. aureus* strain were determined as described by Plowgian et al., with some modification (Plowgian et al. 2019). In brief, *S. aureus* cells were cultured to mid-log phase in Mueller–Hinton broth (MHB) medium. Then the suspensions were centrifuged (4000 rpm for 5 min) and the pellets were collected and diluted to a final concentration of 1 × 10^10^ CFU/mL with MHB. A volume of 100 μL of samples were plated on MHA medium containing NZX with concentrations of 1–256 × MIC (twofold dilution). All plates were incubated at 37 $^\circ{\rm C}$ for 72 h and the concentration corresponding to the plate with no bacterial growth was regarded as the provisional mutant prevention concentration (MPCpr). Additionally, the MPCs values were determined by a linearly decreased concentration at a rate of 20% from the MPCpr. Lincomycin and ceftiofur were set as antibiotic controls, respectively. Each concentration was conducted three times.

### In vitro dose-killing curves

The in vitro dose-killing curves of NZX against the test *S. aureus* strains were conducted as previous described (Brinch et al. 2010), with some modification. The *S. aureus* cells in mid-log phage were diluted. Then, the killing curves of NZX against *S. aureus* strains were carried out in the 48-well plate. Specifically, a volume of 50 μL NZX (0.6–150 μM) was mixed with 450 μL of the above diluted bacterial suspension (5 × 10^5^ CFU/mL), and the mixtures were incubated in the Micro Oscillator for 24 h (600 rpm, 37 °C). Subsequently, the bacteria suspensions were plated on MHA medium to count. Lincomycin and ceftiofur were set as antibiotic controls, respectively. PBS was negative control. Results were expressed as the average of three independent experiments data.

### Post antibiotic effect (PAE)

PAE was performed based on the method of Zhang et al. (Zhang et al. 2014). *S. aureus* E48, *S. aureus* ATCC 25923 and *S. aureus* ATCC 43300 were cultured at 37 °C in MHB medium to the log-phase. Then, the diluted cells (1 × 10^8^ CFU/mL) were exposed to NZX with final concentration of 1 × , 2 × , 4 × and 8 × MIC in the 48-well plates for 2 h at 37 °C. After incubation, NZX were removed by diluting 1:1000 with MHB medium. Viable counts were determined immediately after drug reconstruction and at 1–2 h interval for at least 10 h. PAE determination was calculated as PAE (h) = T-C, where T is the time (h) that required to increase by tenfold for the drug-treated viable count from the moment of drug reconstruction and C is the time (h) required to increase by tenfold for cell density without treatment. The experiments were conducted in triplication.

### Antibacterial mechanism of NZX

#### Interaction of NZX with the S. aureus cell membrane

Flow cytometry was used for the analysis of the effects of NZX on the penetration rate of the *S. aureus* cell membrane (Yang et al. 2019). The *S. aureus* cells in mid-log phase were adjusted to 1 × 10^8^ CFU/mL and the bacteria suspensions were treated with 1 × , 2 × and 4 × MIC of NZX for 2 h at 37 °C. In addition, the cell suspensions were incubated with 2 × MIC of NZX for 0 h, 0.5 h, 1 h and 2 h, respectively. After incubation, the cells were centrifuged at 4000 rpm at 4 °C for 5 min, washed twice with sterile PBS, and pellets were resuspended in 1 mL of PBS. Next, cells were stained with 50 µg/mL propidium iodide (PI) for 30 min in the dark at room temperature, and the proportion of cells with PI positive cells to the total cells were analyzed by flow-cytometry (BD, USA). Lincomycin and ceftiofur were used as positive controls and PBS as negative control. Three replicates for each treatment were conducted.

The cell morphology of *S. aureus* treated with NZX was observed by scanning electron microscope (SEM, QUANTA200, FEI, Philips, Netherlands) (Yang et al. 2019). In detail, *S. aureus* cells (1 × 10^8^ CFU/mL) in log-phase were treated with 4 × MIC of NZX at 37 °C for 2 h, and the cells were centrifuged and washed as described above, after which the pellets were resuspended and fixed with 2.5% glutaraldehyde overnight at 4 °C. The fixed samples were washed three times with PBS to remove fixative and dehydrated for 15 min by a series of ethanol (50% ~ 70% ~ 85% ~ 95% × 2 ~ 100% × 2). Then, the samples were dried by CO_2_ and coated with platinum before the analysis of SEM.

### Super-resolution microscope (SRM) observation

The localization of NZX in *S. aureus* cells was observed by a super-resolution microscope (SRM) (Wu et al. 2022). The *S. aureus* cells (1 × 10^8^ CFU/mL) in log-phase were incubated with 4 × MIC of FITC-NZX and FITC for 30 min at 37 °C. After incubation, cells were washed twice, then incubated with 10 $\upmu$g/mL of DAPI and PI in the dark for 15 min at 4 °C. Then, the suspensions were washed twice again, and the pellets were resuspended in 50 µL of PBS. Subsequently, the samples were transferred to a poly-l-lysine coated glass slides, added anti-fluorescence quenching agent, sealed by nail polish, and finally analyzed using a SRM (N-SIMS, Nikon, Japan).

### Pharmacodynamics of NZX in a mouse mastitis model

#### Mouse mastitis model of infection

The 7 to 8-weeks old ICR mice (80 females, 40 males) were housed in independent ventilated cages, and feed with autoclaved laboratory food and water. Female mice in heat were put into the cage of males (females:males, 2:1) overnight and checked for vaginal plugs at the following morning to induce pregnancy. ICR-lactating mice were used at 6 to 8 days after the birth of the offspring. The mouse mastitis model was established as previously described (Li et al. 2017). Mice were randomly divided into nine groups: NC group (uninfected group), PC group (infected but without treatment), NZX groups (25, 50, 100, 200 and 400 $\upmu$g/gland, respectively), lincomycin group (100 $\upmu$g/gland), and ceftiofur group (100 $\upmu$g/gland). Each experimental group has six independent experiments with three mice. Specially, the pups were removed 1 h before the intramammary injection of both L4 (on the left) and R4 (on the right) abdominal mammal glands. A 100 $\upmu$L of *S. aureus* strains (1 × 10^6^ CFU/mL) was administrated to mice in PC, NZX, lincomycin and ceftiofur groups, and the same volume of sterile physiological saline was administrated to mice in NC group. Glands of 3 h post-infection were injected with sterile physiological saline, NZX, lincomycin, or ceftiofur. The mice were euthanized after the treatment for 24 h, and their mammary glands were dissected, weighed, and homogenized. The animal experiments were performed according to the Animal Care and Use Committee of the Feed Research Institute, Chinese Academy of Agricultural Sciences (Permit Number: AEC-CAAS-20090609) in accordance with the Guidelines for Animal Experimentation.

#### Bacterial counts and histopathological analysis

After mouse sacrifice, one part of mammary tissue was taken for bacterial count, and the other part was used for the histopathological analysis. Specifically, tissues were aseptically collected immediately from the dead mice, weighted, homogenized with sterile physiological saline (1:1, W:V). Next, the homogenates were serially diluted (ten-fold dilutions) in physiological saline, plated on MHA plate, and then cultured overnight at 37 °C. The bacterial number on each plate was counted and the results were expressed as Log_10_ CFU per gram of mammary tissues. In addition, mammary tissues were fixed in 4% paraformaldehyde, then embedded in paraffin, cut into 5 μm sections, and later stained by hematoxylin–eosin (H&E). The stained sections were observed under a light microscope (× 200) for pathological morphology and scoring of mammary tissues (Schmidt et al. 1992).

### Pharmacokinetics of NZX in mouse mammary gland

#### Determination of NZX in mammary tissues and milk

A 0.5 g of mammary tissue or milk were weighed, homogenized, and transferred to a 10 mL polypropylene centrifuge tube, respectively, extracted sequentially by adding into a 3 mL of 0.1 M of McFarland buffer and 1% trichloroacetic acid (TCA), and cleaned by a C18 solid-phase extraction (SPE) cartridge (Oasis MAX 3 cc 60 mg and Oasis HLB 3 cc 60 mg, respectively, Waters, USA). Afterwards, a 2 mL of 45% acetonitrile with 2% formic acid and 3 mL of 45% acetonitrile were used to elute the columns, respectively, the collected eluents were freeze-dried and dissolved in 200 µL of deionized water and filtered by a 0.22 μM sterile filter membrane before the analysis of high-performance liquid chromatography-ultraviolet (HPLC–UV). The HPLC–UV with a C18 reverse-phase column (ZORBAX Eclipse Plus C18 4.6 mm × 250 mm, 5 µm) conditions for detecting NZX were as follows: the mobile phase consisted of A: 0.1% trifluoroacetate (TCA) in acetonitrile and B: 0.1%TFA in water; the injection volume was 40 μL; the wavelength of UV detector was 280 nm; flow rate was 1.0 mL/min; the column temperature was 35 °C, and the elution was conducted with a linear gradient of 20–45% mobile phase A for 16 min. The linear relationship, limits of detection (LOD), limits of quantitation (LOQ), accuracy and precision were determined by adding the known concentrations of NZX into the extracted blank matrix of mammary gland. Each concentration was conducted three times.

#### Stability of NZX in mammary gland and milk environment

A 100 $\upmu$g/mL of NZX was suspended in mammary gland tissue homogenate or milk, respectively, and then incubated at 37 °C for 0, 8, 16 and 24 h, respectively. Afterwards, samples were pretreated and quantified as the above methods. The results were expressed as a ratio of the mean peak area measured at different times divided by the mean value of the initial value.

#### Pharmacokinetics parameters

A pharmacokinetic assessment of a single dose of NZX at 100 $\upmu$g/gland was carried out using 36 individual healthy lactating mice following intramammary administration (Wang et al. 2021). Mammary tissues were harvested at 0.083, 0.5, 1, 2, 3, 4, 6, 9, 12, 16, 20 and 24 h with three mice (six mammary in total) at each time point. The collected samples were then placed into 2 mL eppendorf tubes and stored at − 80 °C before the content determination of NZX in mammary gland by HPLC–UV. Briefly, a portion of the mammary gland tissues (0.5 g) was weighed in 2 mL eppendorf tubes, homogenized for 20 min (50 HZ and 4 °C) in 0.5 mL of PBS solution, and pretreated and purified by a MAX cartridge as described above. The pharmacokinetic parameters of NZX in mammary gland tissues were calculated by non-compartmental model using WinNonlin Phoenix version 8.1 (Pharsight Co., Mountain View, CA, USA).

### Statistical analysis

GraphPad Prism (version 9.5.0) was used for statistical calculations. The statistical significance of differences between each treatment group was compared using one-way ANOVA with Dunnett’s test. A difference with *P*-value < 0.05 was considered as the significant, and different letters represents significant difference (*P* < 0.05) between treatments.

## Results

### In vitro antibacterial characterization

#### MICs, MBCs and MPCs

As shown in Table 1, the MICs of NZX against the three *S. aureus* were 0.23–0.46 μM, which were about 4- to 8-fold lower than those of ceftiofur (1.83–5.49 μM) and 4.7-fold at least lower than those of lincomycin (2.17 ~  > 138.83 μM). The MBCs and MPCs of NZX were all less than those of ceftiofur and lincomycin.

**Table 1:** The MICs, MBCs and MPCs of NZX and antibiotics against *S. aureus* strains (μM)

Antibacterial agents	*S. aureus* E48			*S. aureus* ATCC 25923			*S. aureus* ATCC 43300		
	MIC	MBC	MPC	MIC	MBC	MPC	MIC	MBC	MPC
NZX	0.23^a^	0.23	1.47	0.46^a^	0.46	3.68	0.23^a^	0.92	1.18
Ceftiofur	1.83	3.67	5.49	1.83	3.67	234.24	1.83	1.83	7.32
Lincomycin	2.17	4.34	> 1111.04	2.17	4.34	> 1111.04	> 138.83	> 138.83	-

The value marked by letter a represents that those data had been published by Zheng et al. 2022. “-” indicates that detection wasn’t performed due to a resistant to lincomycin

#### In vitro dose-killing curves

**Fig. 1:**
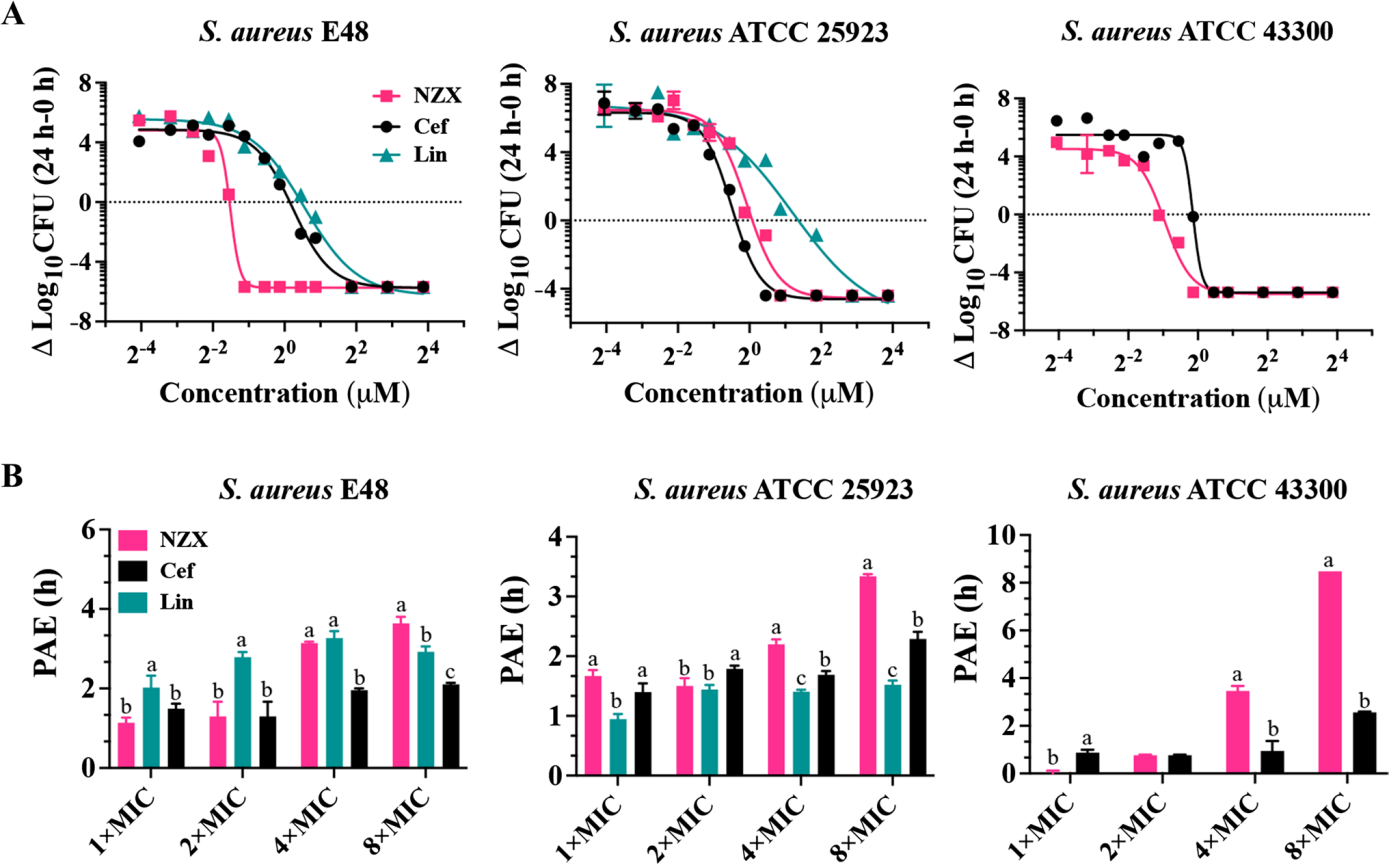
Dose response curves and PAEs of NZX and antibiotic controls against *S. aureus* strains. (A) The dose-killing curves of NZX, ceftiofur and lincomycin against *S. aureus* E48, *S. aureus* ATCC 25923 and *S. aureus* ATCC 43300, respectively; (B) The PAEs of NZX, ceftiofur and lincomycin against *S. aureus* E48, *S. aureus* ATCC 25923 and *S. aureus* ATCC 43300, respectively. Different letters (a–c) on the top of columns represent significant difference (*P* < 0.05) among different treatment groups at the same concentration. Lincomycin and ceftiofur are abbreviated as “Lin” and “Cef”, respectively

The 24 h concentration-responses were examined for NZX in comparison with ceftiofur and lincomycin against three *S. aureus* strains (Fig. 1A and Table 2). For *S. aureus* E48, the maximum effects of NZX were equal to its E_max_ (− 5.73), which showed the similar active to ceftiofur (E_max_: − 5.74) and the more potent than lincomycin (E_max_: − 6.26). The values of EC_50_ and C_s_ for NZX were about 3-to 4-fold lower than those of ceftiofur and lincomycin. For *S. aureus* ATCC 25923, the actual maximal effects for NZX (E_max_: − 4.54) and ceftiofur (E_max_: − 4.61) were observed when their concentration reached a value of 8 µM, but lincomycin (E_max_: − 5.87) wasn’t observed. For *S. aureus* ATCC 43300, the E_max_ for both NZX and ceftiofur all reached their actual maximum bactericidal effects. Considering the values of EC_50_ and C_s_, NZX (EC_50_: 0.52 μM; C_s_: 0.49 μM) appeared to be more potent with value about twofold lower than ceftiofur.

**Table 2:** The corresponding parameters of antibacterial activity of NZX and antibiotics against *S. aureus *in vitro

Strains	Drugs	E_max_ (Log_10_ CFU, 95% CI)	EC_50_	C_s_	*R*^2^
*S. aureus* E48	NZX	− 5.73 (− 6.01 to − 5.45)	0.35	0.34	0.984
	Ceftiofur	− 5.74 (− 6.12 to − 5.38)	1.18	1.11	0.989
	Lincomycin	− 6.26 (− 6.86 to − 5.73)	1.47	1.38	0.986
*S. aureus* ATCC 25923	NZX	− 4.54 (− 5.01 to − 4.09)	0.95	1.06	0.982
	Ceftiofur	− 4.61 (− 4.91 to − 4.33)	0.71	0.77	0.991
	Lincomycin	− 5.87 (− 8.29 to − 4.53)	2.31	2.57	0.966
*S. aureus* ATCC 43300	NZX	− 5.48 (− 5.82 to − 5.16)	0.52	0.49	0.983
	Ceftiofur	− 5.38 (− 5.73 to − 5.03)	0.91	0.91	0.986
	Lincomycin	-	-	-	-

E_max_ is a maximal relative effect (a reduction of Log_10_ CFU at 24 h from the initial inoculum), as an extrapolation for drug concentration at infinitely large concentrations; CI is confidence interval; EC_50_ is regarded as the concentration resulting in a 50% reduction in Log_10_ CFU at 24 h between the minimum and the maximum values; C_s_ is the concentration that causes no apparent bacterial growth in Log_10_ CFU at 24 h compared to the initial inoculum. *R*^2^ represents the correlation coefficient. “-” indicates that detection wasn’t carried out

#### PAE

The PAE results were shown in Fig. 1B. After 2 h of exposure to NZX with concentration ranged from 1 × to 8 × MIC, *S. aureus* E48 showed a mean PAE range of 1.14–3.60 h. In the same conditions, the PAE values of ceftiofur and lincomycin were 1.30–2.10 h and 2.02–3.27 h, respectively. For *S. aureus* ATCC 25923, the PAE of NZX at the same concentrations range was 1.67–3.34 h, and those of ceftiofur and lincomycin were 1.40–2.29 h and 0.94–1.52 h, respectively. When the concentration was increased to 4 × –8 × MIC, NZX had higher PAE values. In addition, the PAE range of NZX against *S. aureus* ATCC 43300 under the same concentration as the above was 0.00–8.48 h, which had a higher PAE value than that of ceftiofur with a mean range of 0.82–1.76 h.

### Antibacterial mechanism of NZX

#### Cell membrane permeability

**Fig. 2:**
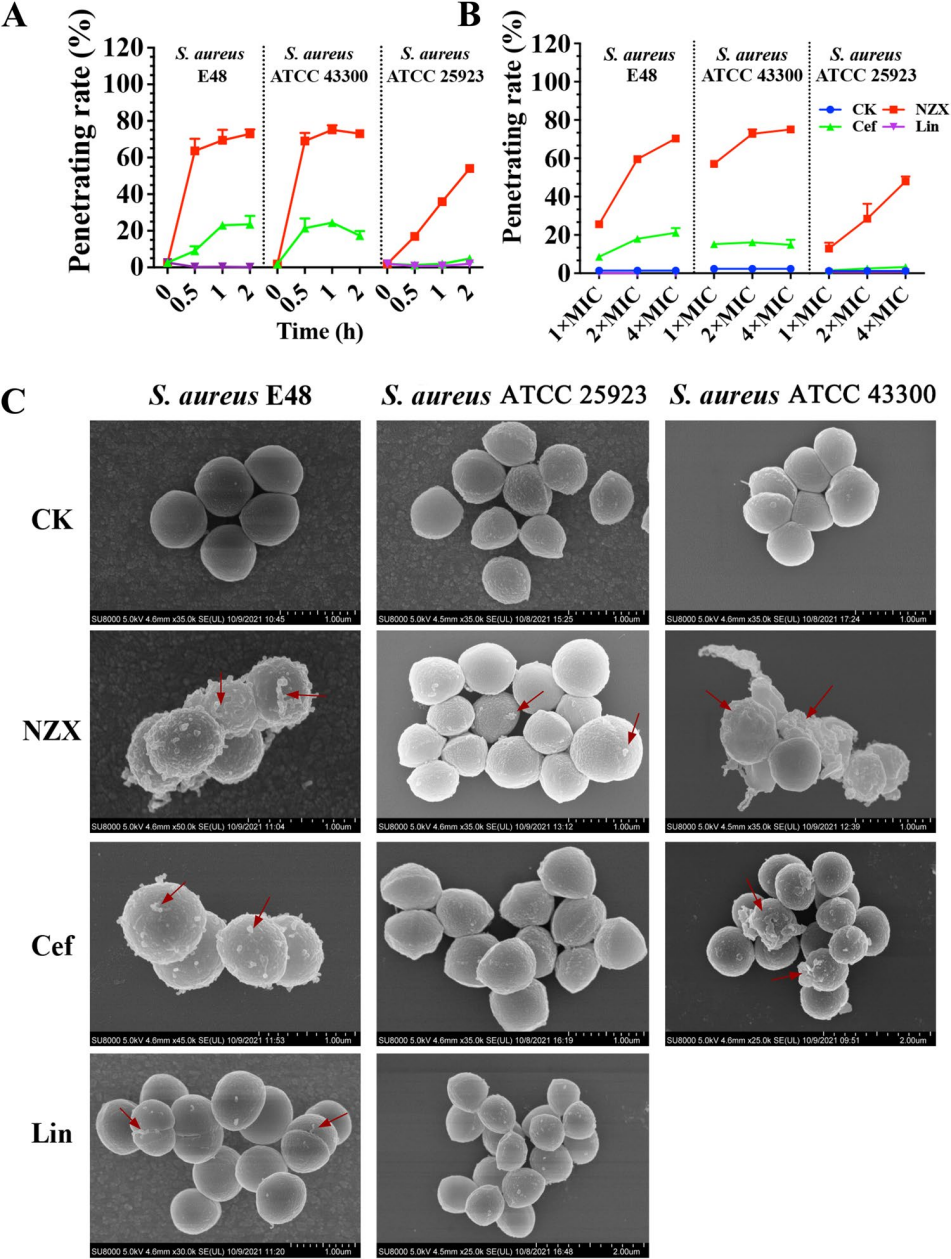
The effects of NZX on cell membrane of *S. aureus*. The penetration rate of NZX, ceftiofur and lincomycin against *S. aureus* ATCC 25923, *S. aureus* ATCC 43300 and *S. aureus* E48 at different time intervals (0 h, 0.5 h, 1 h and 2 h, respectively) (A) and dosage (1× MIC, 2 × MIC and 4 × MIC, respectively) (B), were analyzed by flow cytometer; the cell morphological changes of NZX, ceftiofur and lincomycin at concentration of 4 × MIC against *S. aureus* E48, *S. aureus* ATCC 25923 and *S. aureus* ATCC 43300 (left to right) (C) were observed by SEM. CK represents the negative control; Formations of cell shrinking, membrane shedding and bubble-like structure (indicated by red arrows) were visible

The flow cytometry results were shown in Fig. 2A–B. The percentage of PI-positive *S. aureus* E48 cells ranged from 2.61 to 72.93% after treatment with 2 × MIC for different time intervals (0, 0.5, 1 and 2 h), which was higher than those of ceftiofur (2.61–23.53%) and lincomycin (0.13–2.61%) treatment (Fig. 2A). However, the percentage of PI-positive cells ranged from 1.93 to 54.06% in *S. aureus* ATCC 25923, 1.91 to 75.17% in *S. aureus* ATCC 43300 following treatment NZX, which were all significantly higher than those of ceftiofur (1.35–4.31% and 1.82–24.20%, respectively) and lincomycin (0.79–1.91%) treatment (Fig. 2A). In addition, after treatment with NZX for 2 h at different concentration (1 × MIC, 2 × MIC and 4 × MIC), PI-positive cells of 25.6–70.3%, 12.9–48.0% and 57.1–75.1% were observed for *S. aureus* E48, *S. aureus* ATCC 25923 and *S. aureus* ATCC 43300, respectively (Fig. 2B). And the percentage of PI-positive cells treated with ceftiofur were 8.56–21.1%, 1.55–3.42% and 15.5–15.8%, and ranged from 0.08 to 0.09% and 0.45 to 0.63% after treatment with lincomycin for *S. aureus* E48 and *S. aureus* ATCC 25923, respectively (Fig. 2B).

#### Effects of NZX on cell morphology

SEM was used to analyze the effects of morphology on *S. aureus* surface and the results were shown in Fig. 2C. The whole surface was very smooth and complete without any destruction in the untreated cell control (CK group). Compared with the CK group, the three *S. aureus* cells subjected to NZX treatment at 4 × MIC for 2 h showed typical morphological changes such as membrane shedding (*S. aureus* E48), membrane blebbing (*S. aureus* E48 and *S. aureus* ATCC 25923), cell shrinkage and formation of cell debris (*S. aureus* ATCC 43300). Moreover, antibiotics ceftiofur and lincomycin were noted to induce those morphological changes such as membrane blebbing (*S. aureus* E48) and membrane shedding (*S. aureus* ATCC4330, ceftiofur alone), but significant morphological changes wasn’t observed in *S. aureus* ATCC 25923.

#### SRM observation

**Fig. 3:**
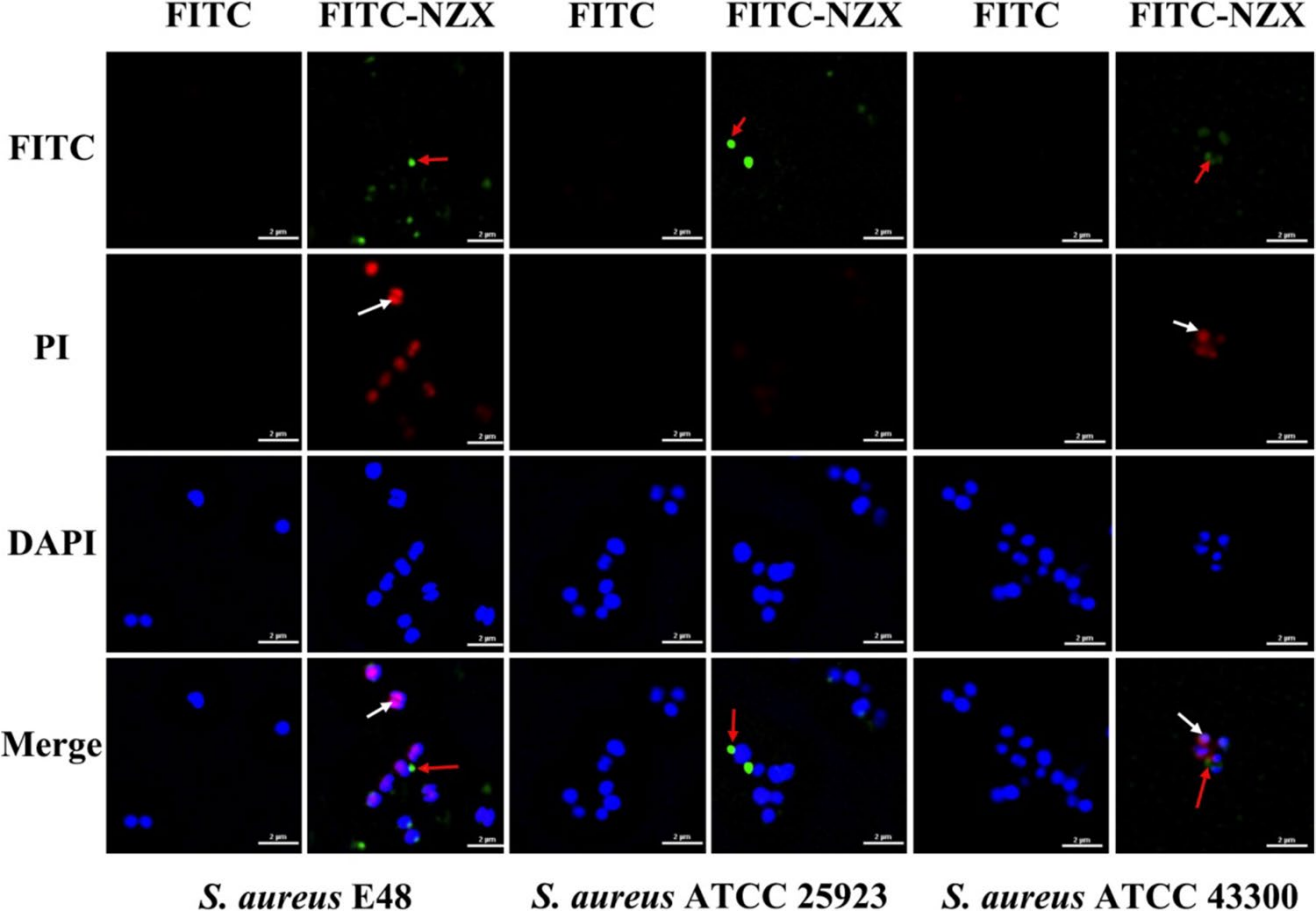
SRM observation of the localization of FITC-labeled NZX in *S. aureus *cells. Green signal (arrow by red), red signal (arrow by white) and blue signal represents FITC-NZX, PI and DAPI, respectively

The localization of NZX in *S. aureus* cells was observed using SRM and the results were shown in Fig. 3. The fluorescence derived from FITC-NZX was highly overlapped with PI in *S. aureus* E48 and ATCC 43300, which indicated that FITC-NZX exerted antibacterial activity by destroying the integrity of the membrane. Meanwhile, the green signal of FITC-NZX surrounded the cell nucleus and co-localized with the blue signal (nuclear dyed of DAPI). But for *S. aureus* ATCC 25923, these phenomena were not observed in the field of vision.

### Pharmacodynamics of NZX in a mouse mastitis model

#### Bacterial counts in mammary tissues

**Fig. 4:**
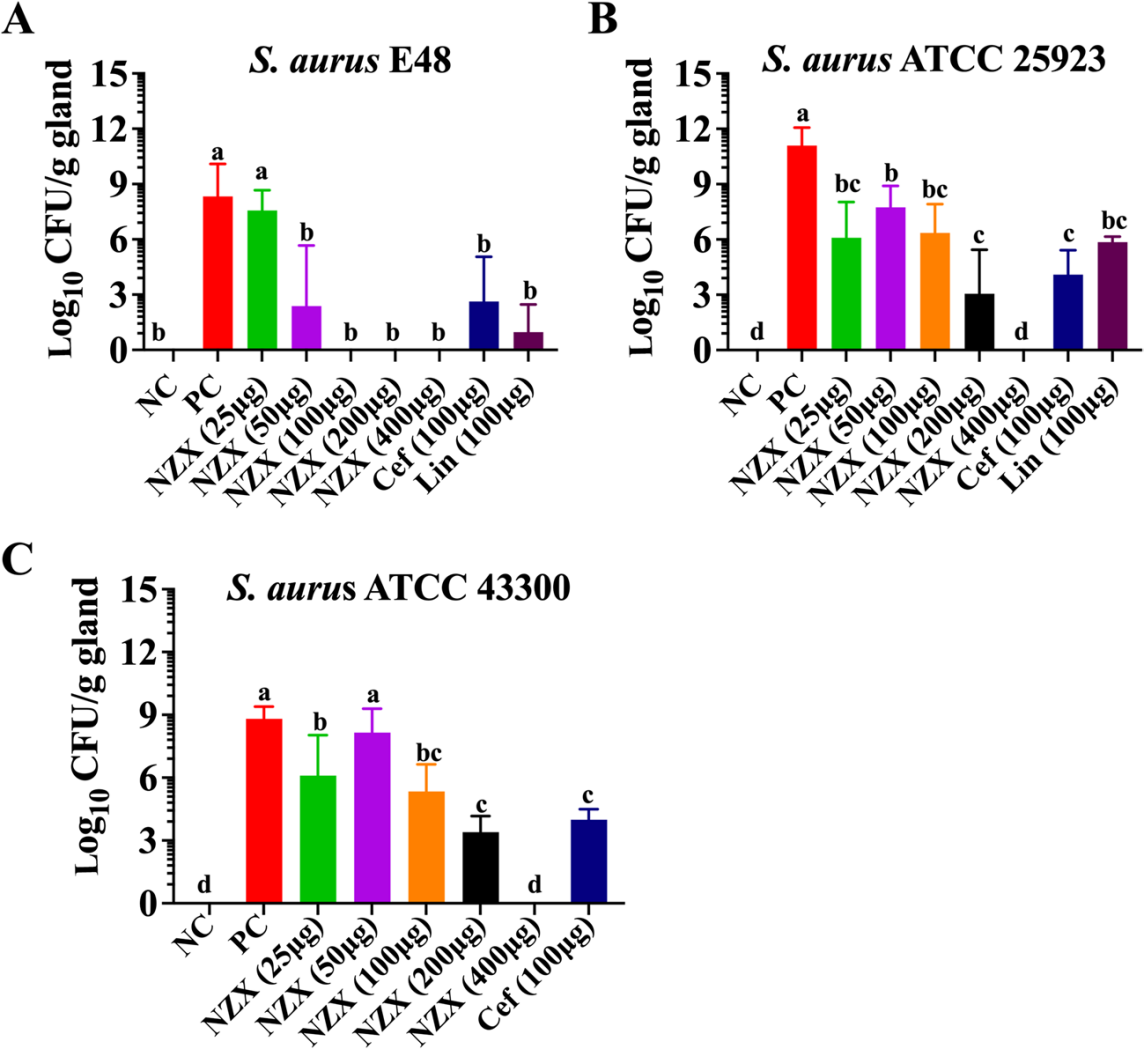
Bacterial loads in mammary tissues of mice. The effects of NZX on bacterial counts in mammary gland infected with *S. aureus* E48 (A), *S. aureus* ATCC 25923 (B) and *S. aureus* ATCC 43300 (C), respectively. NC and PC represent the negative and positive controls; Different letters (a–d) on the top of columns represent significant difference (*P* < 0.05) among different treatment groups. Lincomycin and ceftiofur are abbreviated as “Lin” and “Cef”, respectively

**Fig. 5:**
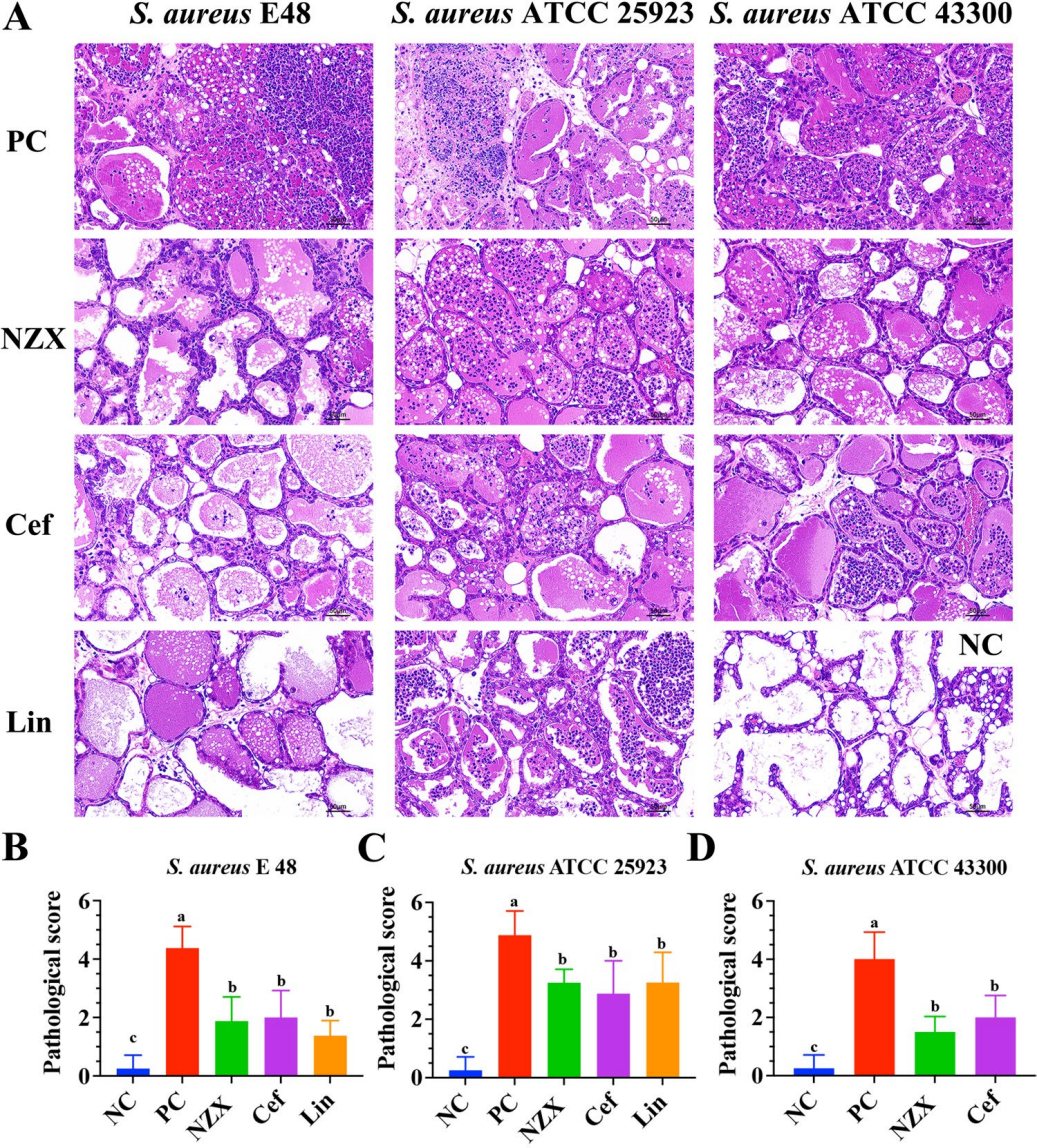
Histopathological observation and scoring of mammary glands in a mouse mastitis model. (A) Histological morphology of mammary tissues treated with sterile physiological saline, NZX, ceftiofur and lincomycin, respectively (Top to bottom); Histopathological scoring of mammary tissues in mouse mastitis model infected with *S. aureus* E48 (B), *S. aureus* ATCC 25823 (C) and *S. aureus* ATCC 43300 (D). “NC” indicates uninfected group; “PC” indicates infected but untreated group. Different letters (a–c) on the top of columns represent significant difference (*P* < 0.05) among different treatment groups

The results of bacterial counts at 24 h after treatment in mouse mastitis model were presented in Fig. 4A–C. After 24 h of infection with *S. aureus* E48, the number of bacteria in mammary tissues reached by 8.34 Log_10_ CFU/g, and the treatment with NZX (25 ~ 400 $\upmu$g/gland) showed a bacteria reduction of 0.77–8.34 Log_10_ CFU/g in a dose-dependent manner (Fig. 4A), and the maximum effectiveness appeared at NZX concentration of 100 $\upmu$g/gland (decreased by 8.34 Log_10_ CFU/g), which was better than those of lincomycin (decreased by 7.37 Log_10_ CFU/g) and ceftiofur (decreased by 5.70 Log_10_ CFU/g) at the same concentration (Fig. 4A). Additionally, the bacterial loads in PC group for *S. aureus* ATCC 25923 increased to 11.09 Log_10_ CFU/g, treatment with 25–400 $\upmu$g/gland of NZX (reduced by 3.35–11.09 Log_10_ CFU/g) also significantly decreased the number of bacteria in a dose-dependent manner (Fig. 4B). NZX with a dosage of 100 $\upmu$g/gland decreased by 4.73 Log_10_ CFU/g after 24 h treatment, which had a lower degree than those of ceftiofur (decreased by 6.99 Log_10_ CFU/g) and lincomycin treatment group (decreased by 5.23 Log_10_ CFU/g), but there were no statistical differences between them (Fig. 4B). When the administration concentration of NZX reached 400 $\upmu$g/gland, the bacterial loads were eliminated, and the best therapeutic effect had achieved (Fig. 4B). In addition, the therapeutic effects of NZX on *S. aureus* ATCC 43300 showed that bacteria number was 8.81 Log_10_ CFU/g in PC group at 24 h post-infection, while NZX (25 ~ 400 $\upmu$g/gland) treatment decreased the number of bacteria by 0.66 ~ 8.81 Log_10_ CFU/g (Fig. 5C). NZX at concentration of 100 $\upmu$g/gland (decreased by 3.47 Log10 CFU/g) showed no significant differences in comparison with that of ceftiofur (reduced by 4.82 Log_10_ CFU/g), while a higher amount of NZX (400 $\upmu$g/gland) was required for a maximum antibacterial effect (reduced by 8.81 Log_10_ CFU/g) (Fig. 4C).

#### Histopathological analysis and scoring in mammary tissues

The changes of histological and mammary tissues scores were shown in Fig. 5A–D. For normal mammary tissue, the structure and appearance of acini were clearly visible, and no lesions were observed in acini and connective tissues (Fig. 5A). However, in mammary tissues from mice infected with the three *S. aureus* strains, there was severe tissue damage observed, particularly characterized by the disappearance of acini, infiltration of inflammatory cell and formation of epithelial cell necrosis. The inflammatory symptoms after the treatment with NZX were obviously improved, but the presence of scattered inflammatory cells in the local stroma of tissues was still observed, and the therapeutic effects of NZX were comparable to those of ceftiofur and lincomycin treatment (Fig. 5A). In addition, results of pathological scores showed that median scores for PC group infected with three *S. aureus* strains were all significantly increased, the scores were significantly decreased after treatment with NZX compared with the PC group, and its effects were equivalent to the treatment of ceftiofur and lincomycin (Fig. 5B–D).

### Pharmacokinetics of NZX in mouse mammary gland

#### Determination of NZX in mammary tissues and milk

**Fig. 6:**
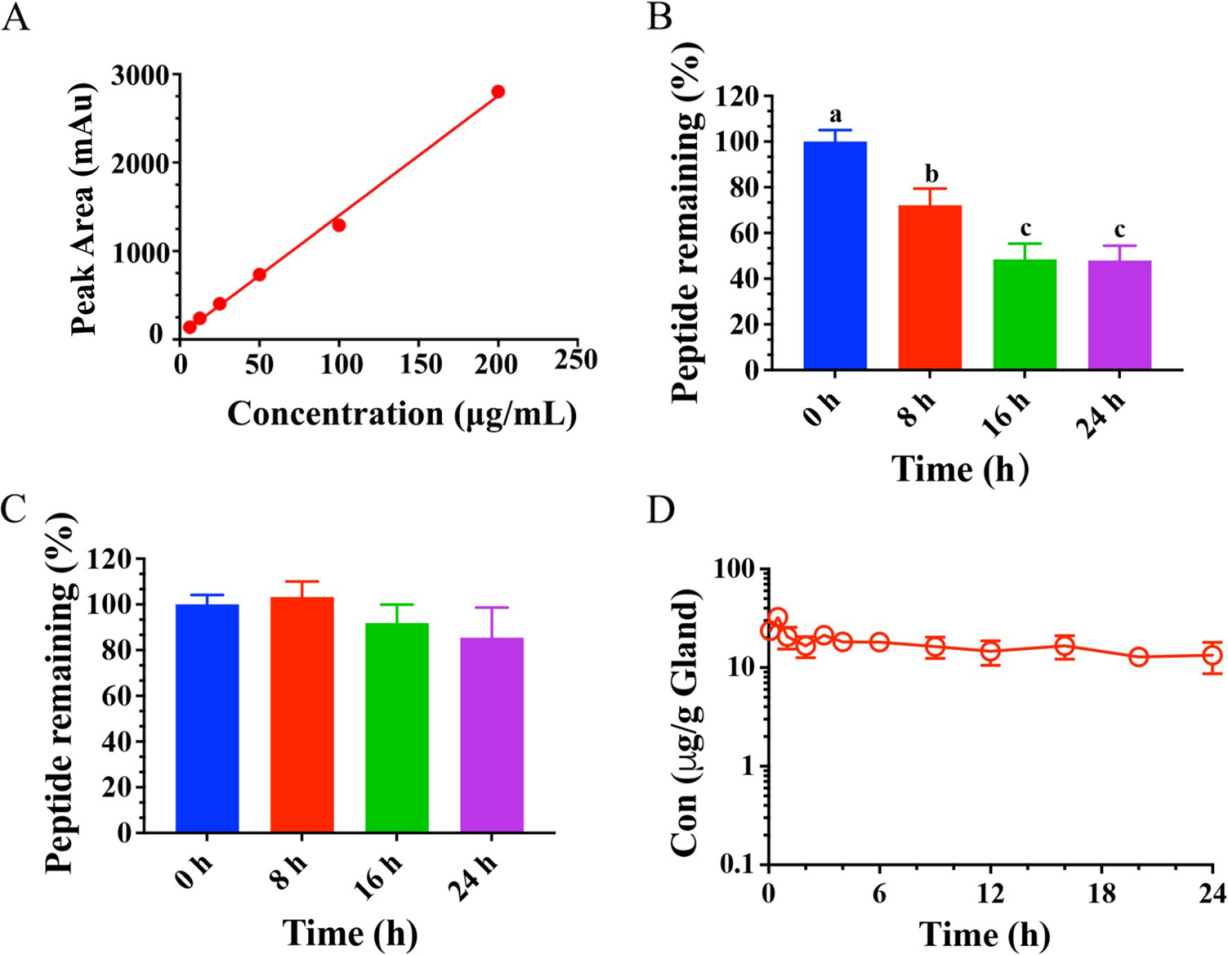
The stability and drug concentration–time curve of NZX. (A) Standard curve of NZX in the blank mammary matrix; The remaining percentage of NZX after incubation with mammary tissue (B) and milk (C) for 0 h, 8 h, 16 h and 24 h, respectively; Different letters represent a significant difference. (D) Drug concentration–time curve of NZX in mouse mammary tissues. Different letters (a–c) on the top of columns represent significant difference (*P* < 0.05) among different treatment time

The results of HPLC method validation show that there was a good linear relationship in the range of 6.25–200 μg/mL (Y = 13.551X + 48.208, *R*^2^ = 0.9969) (Fig. 6A). LOD and LOQ were 1.56 and 3.12 μg/mL, respectively. The average recoveries for 12.5, 25, 50, and 100 μg/mL were 120.27 ± 0.34%, 100.98 ± 2.32%, 103.95 ± 2.26% and 93.66 ± 0.90%, respectively. Furthermore, inter- and intra-day precision for 12.5–100 µg/mL were 0.77–0.98% and 3.76–8.99%, respectively.

#### Stability of NZX in mammary tissue homogenate and milk

Prior to the in vivo study, the stability of NZX in mammary tissues and milk samples were determined. The results showed that residual amount of NZX in the mammary tissue homogenate for 8, 16 and 24 h were 72.19%, 48.43% and 47.90%, respectively, which were significantly decreased in comparison with that of 0 h (100%) (Fig. 6B). In addition, retention rates after the same incubation time in milk were 103.26%, 91.85% and 85.36%, respectively, which showed a slight decreasing trend (Fig. 6C).

#### Pharmacokinetics parameters

The drug concentration–time curve was shown in Fig. 6D. The plot showed that the drug concentration of NZX in mammary tissues after administration had an initial increase followed by a subsequent decrease, and the maximum adsorption of NZX was attained after 0.5 h (T_max_). The estimated parameters in Table 3 showed the peak concentration (C_max_) were calculated as 32.49 μg/g, the terminal phase elimination half-life (T_1/2_) was 35.11 h, area under the concentration time curve (AUC) from time 0 to 24 h after dosing was 391 μg·h/g, respectively.

**Table 3:** Pharmacokinetic parameters of NZX in mammary tissue of mouse by single administration

Parameters	Unites	Estimates
Dose	μg/gland	100
T_1/2_	h	35.11
C_max_	$\upmu$g/g	32.49
T_max_	h	0.5
AUC	$\upmu$g· h/g	391

*T*_*1/2*_, terminal elimination half-life; *C*_*max*_, peak concentration; *T*_*max*_, peak time; *AUC*, area under the concentration time curve from time 0 to 24 h

## Discussion

*S. aureus* is one of the mostly prevalent pathogenic bacteria, which causes recurrent infections in cows leading to serious economic losses to the cow dairy industry worldwide (Cheung et al. 2021; Günther et al. 2017). For a long time, antibiotics have been used as the most common treatment of this disease; however, challenges such as drug resistance, poor intracellular efficiency, and drug residues in milk have emerged (Craft et al. 2019; Dorival-García et al. 2016; Guo et al. 2020; Lehar et al. 2015), it is especially imperative to find new lines of drugs that can reduce the prevalence of *S. aureus*-induced mastitis. Rapid bactericidal action is first advantage of AMPs over traditional antibiotics, which is also one of the reasons why AMPs is not easy to induce bacteria tolerance (Zhang et al. 2022; Buccini et al 2021). Another advantage of AMPs is their less stable and much less environmentally persistent than those of antibiotics, despite the former limits or shortens necessary action period in vivo showing like a double-edged sword (Hao et al. 2023; Zheng et al. 2022). The continuous accumulation of traditional antibiotics in the environment at sublethal concentrations may act as a trigger for resistance evolution (Hao et al. 2023). Additionally, AMPs proved to be potent in inhibiting or eradicating intracellular bacteria (Zheng et al. 2022; Buccini et al. 2021). AMPs, as an ideal alternative therapy, had good properties of rapid killing, low resistance, no residues, and intracellular bactericidal activities, which may be a driving force of a research boom (Buccini et al. 2021). The iron triangle theory of health protection composed of AMPs, antibiotics, and vaccines for keeping the pathogens, antimicrobials, and drug resistances in balance has been recently proposed (Hao et al. 2022). AMPs possess the merits of vaccine and antibiotics and in diseases prevention and treatment, and avoid their risks, such as high resistance and high variation in pathogens, and high residue in animals and environment (Zheng et al. 2022). More to the point, when facing disease, AMPs could move the checkpoint forward, prevent early, improve the basic level of health, treat mild symptoms, reduce the incidence of major diseases and epidemics, avoid high-dose medication for intensive severe symptoms, thus stymie drug resistance at the source.

NZX has good antibacterial activity toward gram-positive bacteria (Liu et al. 2020; Tenland et al. 2018; Wu et al. 2022), suggesting that it may be a viable option in the treatment of gram-positive infections, especially caused by *S. aureus* (MIC: 0.23 ~ 0.46 μM; MBC: 0.23–0.92 μM) (Table 1). MPCs and PAEs were also important parameters to allow antimicrobial dosage regimens to be developed in a much more scientific approach to reduce treatment cost, and the incidences of drug resistant bacteria and avoid unnecessary drug toxicity (Mackenzie and Gould 1993). It had been demonstrated that NZX not only had the potent antimicrobial activity than other plectasin-derived peptide in vitro and in vivo, but also had the lower MPC value (1.82 µM) against *S. agalactiae* when compared with P2 (> 29.2 µM) (Wu et al. 2022). In our study, when it compared to antibiotic control (ceftiofur and lincomycin), the lower MPC values of NZX (1.18–3.68 $\upmu$ M) against *S. aureus* indicated its low probability of resistant mutations. Moreover, PAE values of NZX against *S. aureus* strains displayed the characteristics of strain specific and concentration-dependent differences. Specifically, NZX has longer PAE value against *S. aureus* ATCC 43300 compared to those of *S. aureus* E48 and *S. aureus* ATCC 25923, which was similar to the value of NZ2114 for *S. aureus* ATCC 43300 reported by Zhang et al. (Zhang et al. 2014).

The cell membrane is the initial contact point for most natural AMPs, which can effectively destroy the membrane and induce a leakage of cytosol. It has been demonstrated that plectasin along with its derived peptide could damage the cell membrane and cause cell morphological change of Gram-positive bacteria (Zheng et al. 2021; Yang et al. 2019). However, unlike P2 and plectasin (Yang et al. 2019), NZX had the higher cell membrane penetration rate against *S. aureus*. In addition, serious cell damage of *S. aureus* (expect for *S. aureus* ATCC 25923) after treatment with NZX, such as membrane perforation, membrane shedding, membrane blebbing, cell shrinkage or formation of cell debris, were observed by SEM analysis, which was like those of treatment with ID13 and P2 (Yang et al. 2019; Li et al. 2020). DNA as another intracellular target for AMPs against bacteria was widely studied (Sharma and Nagaraj 2015; Sharma and Khuller 2001). In this study, the results suggested NZX could interact with intracellular DNA of *S. aureus* E48 and ATCC 43300. But For *S. aureus* ATCC 25923, this phenomenon was not observed in the field of vision probably because of low penetration rate of NZ2114 to *S. aureus* ATCC 25923 compared to *S. aureus* E48 and ATCC 43300 and only one fixed time observation point.

Due to the characteristics of nonspecific adsorption, protease sensitivity and high protein binding, AMPs may cause different degrees of loss after sample pretreatment (Sieprawska-Lupa et al. 2004; Starr et al. 2016; Svenson et al. 2007). For instance, Schmidt et al. found that the recovery rates of oncocin analogues Onc72 and Onc112 were 45% and 72% in mouse plasma, 21% and 33% in the brain, and 1.6% and 1.7% in the kidney, respectively (Schmidt et al. 2016). In this study, the extraction conditions and the corresponding recovery rate of NZX in mammary tissues were explored, the results showed that the absolute recovery rate of NZX was 43.58% after pre-precipitation with 0.1 M of McFarland buffer and 1% trichloroacetic acid (TCA), which showed that the two-step protein precipitation is a key to affect the recovery rate. Meanwhile, those issues indicated the course of pre-treatment significantly affects the recovery of peptide and brought the obstacles toward their quantification in biological matrix. Therefore, some researchers had been devoting to the quantitative methods development of peptide that distinguished with small molecular drugs over the past few decades (Andes et al. 2009; Gorman et al. 2010; Wang et al. 2012). Compared to immunoassay and microbiological methods, chromatography method has the merits of good reproducibility, high sensitivity, and wide linear ranges (Ewles and Goodwin 2011; Lei et al. 2018; Zheng et al. 2022). In this study, HPLC method used to determine NZX concentrations in mammary tissues was established, and the method validation results showed that there were no interfering peaks near the target peak, indicating that the method has good specificity. In addition, it showed a good linear relationship and repeatability, and high recovery rate. Stability results of NZX measured by HPLC method showed that it was relatively stable for 24 h in milk environment, but it was degraded to 47.90% in the presence of mammary tissue homogenate, which demonstrated protease in mammary tissues may be one of the important factors affecting its stability.

Due to the high cost and uncontrollable conditions for the studies of the cow mastitis, the pharmacodynamics and pharmacokinetics of new antibacterial drugs using the mouse model had become popular (Wang et al. 2021; Yu et al. 2016). It had been reported that AMPs showed potential in the treatment of mastitis caused by different pathogen bacteria (Li et al. 2017; Yang et al. 2022). In this study, NZX could significantly reduce the bacteria number in mammary gland tissue in a dose-dependent way, and the group with the dosage of 100 $\upmu$g/gland showed the superior or like those group of ceftiofur and lincomycin, which showed a better therapeutic effect than MP1102 and NZ2114 in the treatment of mouse mastitis caused by *S. aureus* E48 (Li et al. 2017). In addition, AMPs not only could play an important role in protecting the host from pathogens by direct bactericidal activity but also through indirect host immune defense. In previous studies, it was demonstrated that the supplementation of AMPs, such as porcine β-defensin 2, sublician, plectasin and NZ2114, could attenuate inflammation or enhance innate immunity response in different hosts (Han et al. 2015; Ma et al. 2020; Li et al. 2021; Zheng et al. 2021). Similarly, the inflammatory symptoms of mammary gland tissues after the treatment with NZX got obvious improved, which was line with the results of NZ2114, MP1102 and P2 (Li et al. 2017; Yang et al. 2022).

The pharmacokinetics of plectasin, NZ2114 and NZX by intravenous injection had been performed (Andes et al. 2009; Brinch et al. 2009; Zheng et al. 2022). Unlike those results conducted by intravenous injection with short half-lives, the pharmacokinetic parameters of NZX (100 µg/gland) administrated by milk duct in mouse mammary gland indicated that it maintained a high concentration (C_max_: 32.49 µg/g) and a longer time (T_1/2_: 35.11 h) in mammary tissues after administration. The isoelectric point of NZX is 8.30, higher than the pH of mouse or cow milk (6.5–6.8) (Yu et al. 2016), it exists in an ionized state under the condition with low fat solubility leading to the difficulty in penetrating the blood-milk barrier, and which may further explain why NZX maintains higher drug concentrations in breast tissue for a longer period. Although NZX level in mammary tissues were consistently higher than MIC, its maximum effectiveness with dosage of 100 $\upmu$g/gland was only found in *S. aureus* E48 and did not find for *S. aureus* ATCC 25923 and *S. aureus* ATCC 43300, which may own to the lowest EC_50_ and C_s_ of NZX against *S. aureus* E48 (0.35 µM and 0.34 µM) than those of *S. aureus* ATCC 25923 (0.95 µM and 1.06 µM) and *S. aureus* ATCC 43300 (0.52 µM and 0.49 µM) (Fig. 1A). In addition, the distribution of NZX in mouse injected by intravenous way found that they could reach kidneys, liver, spleen or lung tissues (Zheng et al. 2022). However, due to the presence of blood-milk barrier, NZX administrated by milk duct was only distributed into mammary gland tissues without detection or observe in serum and organs (data not shown). We consider this work, previous our and other works provide strong support to realize pharmacokinetics and meet the requirement for evaluation of AMPs focusing on mastitis treatment and route of administration by mammary duct injection by combining non-compartment and function pharmacokinetics, which will break through the bottleneck of AMPs for enriching the pipeline of new drug development, realize full potential of AMPs’ special function such as intracellular bactericidal action and membrane penetration contributing to effectively treating clinical mastitis and subclinical mastitis in dairy cows. In conclusion, compared to ceftiofur and lincomycin, NZX showed a superior antibacterial activity towards *S. aureus* E48, *S. aureus* ATCC 25923 and *S. aureus* ATCC 43300, and it also exhibited the low incidence of resistant mutations. In addition, NZX displayed the effect of membrane penetrating and could lead to the obvious morphological changes of *S. aureus* strains (expect for *S. aureus* ATCC 25923). NZX was relatively stable in milk, but it was easily degraded in the presence of mammary tissues homogenate. Moreover, NZX could effectively eradicate bacteria of mammary tissues in dose-dependent manner and significantly decrease the pathological scoring, which was comparable to those of ceftiofur and lincomycin. The drug concentration–time curve of NZX in mouse mammary gland was obtained and the corresponding parameters of T_1/2_, C_max_ and AUC were 35.11 h, 32.49 μg/g and 391 μg·h/g, respectively. In conclusion, NZX, as a safety and effective antibacterial agent, has a great potential for clinical treating dairy mastitis disease caused by *S. aureus*.

## Data Availability

All data generated or analyzed during this study are included in this published article.
